# Multidrug resistant AmpC β-lactamase producing *Escherichia coli* isolated from a paediatric hospital

**DOI:** 10.12669/pjms.301.4045

**Published:** 2014

**Authors:** Noor-ul-Ain Jameel, Hasan Ejaz, Aizza Zafar, Hafsa Amin

**Affiliations:** 1 Noor-ul-Ain Jameel, (M.Phil), Institute of Molecular Biology and Biotechnology, The University of Lahore, Lahore, Pakistan.; 2 Hasan Ejaz, (M.Phil), Department of Microbiology, The Children’s Hospital and Institute of Child Health, Lahore, Pakistan.; 3 Aizza Zafar, (M.Phil), Department of Microbiology, The Children’s Hospital and Institute of Child Health, Lahore, Pakistan.; 4 Hafsa Amin, (M.Phil), Institute of Molecular Biology and Biotechnology, The University of Lahore, Lahore, Pakistan.

**Keywords:** *E. coli*, AmpC β-lactamase, Antimicrobial resistance, Multidrug resistant *E. coli*

## Abstract

***Objective*** *:* The objective of the study was to observe the antimicrobial resistance of AmpC β-lactamase producing *E. coli.*

***Methods:*** Six hundred and seventy *E. coli *were isolated from 20,257 various pathological samples collected from The Children’s Hospital and Institute of Child Health, Lahore, Pakistan. The isolates showed resistance to ceftazidime which were further examined for AmpC β-lactamase activity by Disc Potentiation method.

***Results:*** There were 670 isolates of *E. coli *out of which 85 (12.6%) were AmpC β-lactamase producers. Risk factors like intravenous line (76.5%), endotracheal tube (22.4%), surgery (12.9%) and urinary catheters (7.1%) were found to be associated with infection caused by AmpC β-lactamase producing *E. coli. *Antimicrobial resistance pattern revealed that AmpC producing *E. coli *were highly resistant to co-amoxiclav, ceftazidime, cefotaxime, cefuroxime, cefixime, ceftriaxone and cefoxitin (100% each). Least resistance was observed against sulbactam-cefoperazone (14.1%), cefepime (7.1%), piperacillin-tazobactam (5.9%) and none of the isolates were resistant to imipenem and meropenem.

***Conclusion:*** The minimum use of invasive devices and strict antibiotic policies can reduce the spread of AmpC β-lactamase producing *E. coli. *

## INTRODUCTION

β-lactam antibiotics account for approximately 50% of global antibiotic consumption which has considerably increased the resistance in Gram negative bacteria.^1^ AmpC β-lactamase production is one of the commonest causes of resistance to β-lactam antibiotics among Gram-negative bacteria. AmpC β-lactamases are resistant to aminopenicillins, carboxypenicillins, ureidopenicillins, cephalosporins, broad as well as extended spectrum cephalosporins (cephamycin) and monobactams (aztreonam).^2^^,^^3^ AmpC β-lactamases are resistant to β-lactamase inhibitors like clavulanic acid.^4^

*E. coli* is a major organism among normal flora and it causes a wide variety of intestinal and extra-intestinal diseases, such as diarrhea, urinary tract infections, septicemia and neonatal meningitis.^5^ It is resistant to a wide variety of clinically important antibiotics due to production of AmpC β-lactamase enzyme.^6^ Most of the risk factors of AmpC producing *E. coli *infections include prolonged hospital and intensive care unit stay, use of urinary, arterial or venous catheters, ventilator assistance, haemodialysis, emergency abdominal surgeries, use of naso gastric tube and prior use of β-lactamase antibiotic.^7^^,^^8^

Clinical isolates of AmpC β-lactamase producing *E. coli* and their antimicrobial resistance have been described from different parts of the world.^9^^-^^11^ However, there are only few studies from Pakistan, which have systematically reported the role of various interventions and antimicrobial resistance of AmpC β-lactamase producing *E. coli. *This study was undertaken to assess the risk factors and antimicrobial resistance pattern of such *E. coli *isolated from paediatric patients.

## METHODS

This study was conducted at Microbiology Department of The Children’s Hospital and Institute of Child Health Lahore, Pakistan, during March 2011 to February 2012. A total number of 670 *E. coli *strains were isolated from various clinical specimens such as blood, pus, urine, sputum, tracheal secretions and various tips. The isolates were identified as *E. coli* by colonial morphology, Gram’s stain, catalase test, oxidase test and API 20E system (bioMerieux, France).^12^

 Isolates were screened for AmpC β-lactamase production by disc diffusion method as described by Clinical Laboratory Standards Institute (CLSI).^13^ The *E. coli *which showed reduced susceptibility to ceftazidime and cefotaxime were selected for further confirmation by Disc Potentiation method using 3-amino phenyl boronic acid (APB).^14^

A suspension of each isolated AmpC β-lactamase producing *E. coli* was made according to the 0.5 McFarland turbidity standard and antimicrobial susceptibility testing was performed using two plates on Mueller Hinton agar (90mm) for each strain. The antibiotic discs of amikacin (30 µg)*, *aztreonam (30 µg), cefepime (30µg), cefixime (5 µg)*, *cefotaxime (30 µg)*, *cefoxitin (30 µg)*, *cefpodoxime (30 µg)*, *ceftazidime (30 µg)*,*cefuro xime (30 µg)*, *ciprofloxacin (5 µg)*, *co-amoxiclav (20/10 µg)*, *co-trimoxazole (1.25/23.75 µg)*, *gentamycin (10 µg)*, *meropenem (10 µg)*, *imipenem (10 µg)*,* piperacillin-tazobactam (100/10 µg) and sulbactam-cefoperazone (75/30 µg) were placed on Mueller-Hinton agar plates and incubated overnight at 37℃. After overnight incubation the diameter of each zone of inhibition was measured in mm. The antimicrobial susceptibility testing results were noted according to the CLSI guidelines.^13^

The clinical record of each patient was reviewed. The patients were assessed for the various interventions like intravenous line, endotracheal tube, surgery, peritoneal dialysis catheters, nasal gastric tube, urinary catheters and central venous pressure line.

## RESULTS

 During the study period, 20,257 clinical samples were processed for isolation of AmpC β-lactamase producing *E. coli*. Out of 670 *E. coli *isolated from these samples*, *there were 85 (12.6%) AmpC β-lactamase producers.

The 85 patients infected with AmpC producing *E. coli *had undergone through various interventions during hospitalization as shown in Table-I. These interventions included intravenous lines 65 (76.5%), endotracheal tubes 19 (22.4%), surgeries 11 (12.9%), peritoneal dialysis catheters 8 (9.4%), naso gastric tubes 6 (7.1%) and central venous pressure lines 2 (2.4%).

All the 85 (100%) AmpC producing *E. coli *were resistant to co-amoxiclav, ceftazidime, cefotaxime, cefuroxime, cefixime, ceftriaxone and cefoxitin. AmpC producing *E. coli *showed less resistance to sulbactam-cefoperazone 12 (14.1%), cefepime 6 (7.1%) and piperacillin-tazobactam 5 (5.9%). None of the isolates were found to be resistant to imipenem and meropenem (Table-II).

## DISCUSSION

The emergence of resistance to the third generation cephalosporins in Gram negative bacteria is a major concern which is mostly caused by AmpC β-lactamase. It is difficult to treat multidrug resistant AmpC β-lactamase producing *E. coli*. High frequency of AmpC β-lactamase producing *E. coli *and their resistance to antibiotic has been reported in many areas of the world and which is continuously increasing.^2^ In our study, 12.6% of AmpC producing *E. coli *were isolated from paediatric patients. These observations are similar to the studies carried out by some other workers.^15^^,^^16^ Generally, hospital environment accounts high number of resistance bacteria which frequently transfers from one patient to another.

**Tables-I:** Various interventions among AmpC positive *E. coli *patients (n=85).

*Interventions*	*AmpC positive E. coli*	
	*n*	*%*
Intravenous line	65	76.5
Endotracheal tube	19	22.4
Surgery	11	12.9
Peritoneal dialysis catheter	8	9.4
Naso gastric tubes	6	7.1
Urinary catheters	6	7.1
Central venous pressure line	2	2.4

**Table-II:** Antimicrobial resistance of AmpC β-lactamase producing *E. coli.*

*Antibiotics*	*Resistant n (%)*
Co-amoxiclav (20/10µg)	85 (100)
Ceftazidime (30µg)	85 (100)
Ceftriaxone (30µg)	85 (100)
Cefotaxime (30µg)	85 (100)
Cefixime (5µg)	85 (100)
Cefuroxime (30µg)	85 (100)
Cefoxitin	85 (100)
Co-trimoxazole (1.25/23.75µg)	78 (91.8)
Cefpodoxime (30µg)	74 (87.1)
Aztreonam (30µg)	59 (69.4)
Gentamicin (10µg)	53 (62.4)
Amikacin (30µg)	52 (61.2)
Ciprofloxacin (5µg)	29 (34.1)
Sulbactam-cefoperazone (75/30µg)	12 (14.1)
Cefepime (30µg)	6 (7.1)
Piperacillin-tazobactam (100/10µg)	5 (5.9)
Imipenem (10µg)	0 (0)
Meropenem (10µg)	0 (0)

 There are many factors such as various interventions during hospitalization which are associated with the transmission of AmpC β-lactamase producing bacteria. In our study various such interventions were intravenous lines (76.5%), surgeries (12.9%), peritoneal dialysis catheters (9.4%), naso gastric tubes (7.1%), urinary catheters (7.1%) and central venous pressure lines (2.4%). The risk factors associated with AmpC producing organism have also been investigated in different studies. A case control study on AmpC β-lactamase producing *E. coli *was carried out among the patients who had undergone various invasive procedures who had bacteremia. These included urinary catheter (37%), peritoneal dialysis catheter (6.3%) and intravenous lines (3.7%).^17^ Another study reported indwelling urinary catheter (25.9%) and central venous catheter (29.6%) as risk factors for infections caused by AmpC β-lactamase producing strains.^18^ These findings suggested that these risk factors posed a threat for the patients to become colonized or infected with AmpC β-lactamase producing strains. The patients who receive these interventions like intravenous line, urinary catheters and other catheters become susceptible to infections caused by AmpC β-lactamase producing strains.

In the current study, AmpC β-lactamase producing *E. coli* were multidrug resistant. All were resistant to co-amoxiclav, ceftazidime, cefotaxime, ceftriaxone, cefixime, cefuroxime and cefoxitin. These findings are in accordance with the work done by some researchers. One such study from Korea reported all of the AmpC producing *E. coli *were resistant to co-amoxiclav, ceftazidime, cefotaxime, ceftriaxone and cefoxitin (100% each).^19^ Similar observations were found in another study from Spain.^20^ These findings clearly show that AmpC β-lactamase producing *E. coli *strains are highly resistant to clinically important antibiotics. Continuous or frequent use of these antibiotics probably leads to higher resistance rates of AmpC-producing isolates, especially in paediatric populations.^15^

The isolated AmpC β-lactamases producing* E. coli* found to be significantly resistant to co-trimoxazole (91.8%), cefpodoxime (87.1%), aztreonam (69.4%), gentamicin (62.4%), amikacin (61.2%) and ciprofloxacin (34.1%). These findings are slightly different from other studies. One study conducted in China observed antibiotic resistance of AmpC β-lactamase producing *E. coli* from different paediatric hospitals. The isolated strains were found to be resistant to ciprofloxacin (70%), gentamicin (70%) and amikacin (30%).^15^ Another study carried out in France reported that AmpC β-lactamase producing *E. coli* isolated from bacteremic patients were considerably resistant to ciprofloxacin (50%) but less resistant to gentamicin (5.6%) and none of the strain was resistant to amikacin.^21^ In another study from Korea, none of the AmpC β-lactamase producing *E. coli* showed resistant to co-trimoxazole, aztreonam, cefpodoxime, gentamicin, amikacin and ciprofloxacin.^19^ Higher rates of resistance to these antibiotics in our study could also be due to other possible mechanisms like efflux pump or loss of porin. 

AmpC β-lactamase producing *E. coli* were found to be less resistant to sulbactam-cefoperazone (14.1%), cefepime (7.1%) and piperacillin-tazobactam (5.9%) in our study. Contrary to our results, studies from Korea and Canada reported none of AmpC β-lactamase producing *E. coli* resistant to sulbactam-cefoperazone, cefepime and piperacillin-tazobactam.^19^^,^^22^ Mulvey et al reported, 4.7% of 65 AmpC β-lactamase producing *E. coli *showed resistance to piperacillin-tazobactam.^16^

None of the AmpC β-lactamase producing *E. coli *was found resistant to imipenem and meropenem in our study. Similar findings have been reported in other studies conducted in Japan, United States and Spain.^20^^,^^23^ A study from Pakistan reported resistance of AmpC producing bacteria to gentamicin (75%), ciprofloxacin (75%), amikacin (65%) and sulbactam-cefoperazone (32.5%) and none of strain was found resistant to meropenem.^24^ These finding suggest that imipenem and meropenem might be useful for the treatment of infections caused by AmpC β-lactamase producing organisms.

Thus meropenem, imipenem, piperacillin-tazobactam, cefepime and sulbactam-cefoperazone could be drugs of choice for treating AmpC β-lactamase producing *E. coli* infections. The burden of AmpC producing *E. coli *strains can be reduced by minimizing the use of invasive devices and strict adherence of antibiotic policy. Communication between the hospitals and the other health institutions regarding the prevalence of resistant bacteria, identifiable risk factors and controlled procedures can decrease the risk of AmpC β-lactamase producing bacteria.
